# Surface Functionalization of Zeolitic Imidazolate Framework‑8 Nanoparticles with Accessible Groups for Covalent Conjugation

**DOI:** 10.1021/acs.inorgchem.6c01777

**Published:** 2026-07-01

**Authors:** Michael B. Stammler, Hana Bunzen

**Affiliations:** 1 Chair of Solid State and Materials Chemistry, Institute of Physics, 26522 University of Augsburg, Universitätsstraße 1, Augsburg 86159, Germany; 2 Chair of Inorganic Chemistry, Institute of Chemistry, Otto-von-Guericke University Magdeburg, Universitätsplatz 2, Magdeburg 39106, Germany

## Abstract

Zeolitic imidazolate framework-8 (ZIF-8) is a widely studied metal–organic framework for a range of applications. However, its limited availability of reactive surface groups restricts controlled covalent functionalization. Here, we introduce amino (–NH_2_), aldehyde (–CHO), and carboxylate (–COOH) functionalities onto nanosized ZIF-8 via solvent-assisted ligand exchange while preserving crystallinity and morphology. The degree of ligand exchange can be tuned by adjusting the reaction time, reaching values between 4 and 15%, as quantified by NMR spectroscopy. The chemical accessibility of the introduced groups is demonstrated through covalent conjugation reactions, including fluorescein labeling and carbodiimide-mediated amide formation. This approach establishes a versatile platform for equipping ZIF-8 nanoparticles with reactive surface functionalities, enabling modular postsynthetic modification for applications in catalysis, sensing, biomedicine, and beyond.

## Graphical abstract

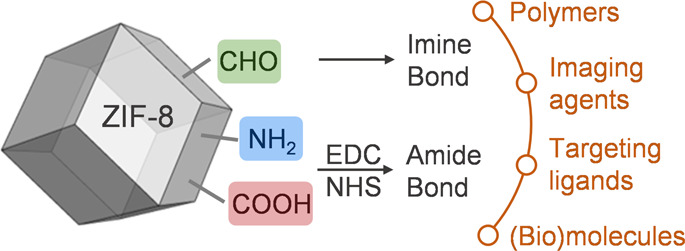

## Introduction

Zeolitic imidazolate framework-8 (ZIF-8),^1^ also known as MAF-4,^2^ is among the most extensively studied metal–organic frameworks (MOFs),^3,4^ particularly for biomedical applications.^5,6^ Structurally, ZIF-8 consists of Zn­(II) ions coordinated by 2-methylimidazolate ligands, forming a sodalite-type framework with large internal cavities (11.6 Å) connected by small pore apertures (3.4 Å).^1^ ZIF-8 has been widely explored as a drug delivery platform for small-molecule chemotherapeutics, biomacromolecules, and nucleic acids.^7–9^ Its mild synthesis conditions enable the *in situ* encapsulation of sensitive molecules, including biomolecules, without significant loss of bioactivity.^10,11^ Furthermore, its relative instability under acidic conditions^12,13^ offers the possibility for pH-triggered drug release.^7,14^ Beyond drug delivery, ZIF-8 has also shown promise in bioimaging and theragnostic applications.^15,16^

For biomedical application, external surface modification of nanoparticles is a critical aspect, as it directly governs interactions with biological environments.^17^ While the intrinsic porosity of MOFs enables high cargo loading, surface chemistry determines colloidal stability in physiological media, cellular uptake, biodistribution, and clearance.^18^ Functionalization with polymers, lipids, biomolecules, or targeting ligands can enhance biocompatibility, reduce nonspecific interactions, prolong circulation time, and enable active targeting of specific cell types or tissues.^18–20^ Furthermore, tailored surface coatings can act as protective barriers, modulating degradation kinetics, preventing premature cargo release, and improving the stability of MOF nanoparticles under complex biological conditions. Precise control over the external surfaces of MOF nanoparticles is therefore essential to bridge the gap between promising *in vitro* performance and reliable *in vivo* functionality and clinical translation.^21,22^

Several strategies have been proposed for attaching various compounds to the external surfaces of MOFs, including noncovalent interactions, coordinate bonds, and covalent bonds.^19,23^ In this work, we focus on covalent attachment. While covalent functionalization has been extensively studied for carboxylate-based MOFs, for example, using the amino group of 2-aminoterephthalate ligands^24^ or benzoic acid capping ligands bearing various functional groups,^25^ studies on covalent modification of the ZIF-8 surface are lacking. This is because the 2-methylimidazolate ligands of ZIF-8 do not contain reactive functional groups that are suitable for conjugation (Figure 1). One alternative is to use ZIF-90, which comprises 2-carboxyimidazolate ligands with aldehyde groups that can be used for conjugation.^26^ However, replacing ZIF-8 with ZIF-90 requires adjustments to the synthesis conditions, and the change in pore polarity may introduce additional challenges, for example, affecting drug loading. Therefore, in this study, we focus on ZIF-8 and explore strategies to introduce functional groups, such as amino, aldehyde, and carboxylic acid groups, onto its external surface, which can be readily used in common (bio)­conjugation reactions (Table 1) to covalently attach selected molecules, including fluorescent dyes, biomolecules, polymers, or targeting ligands.

**Figure 1:**
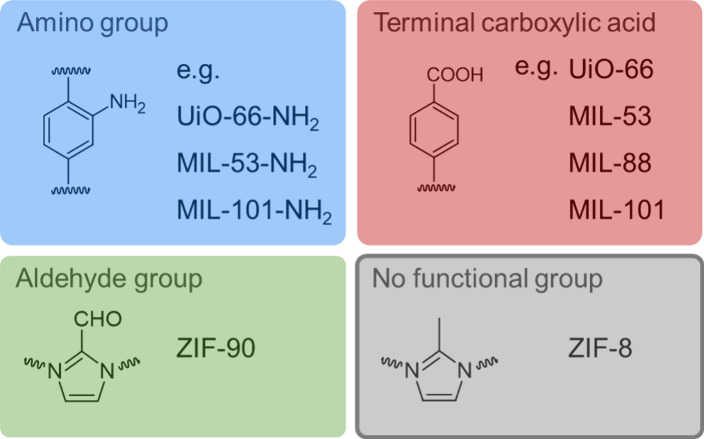
Examples of functional groups in MOFs that are readily available for (bio)­conjugation reactions and the corresponding MOFs.

**Table 1:**
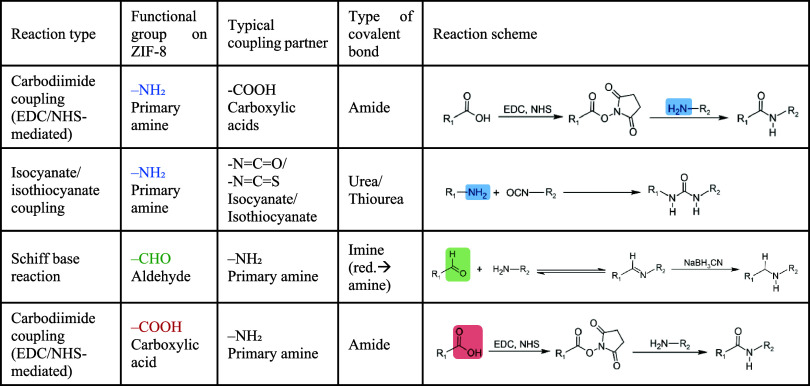
Examples of Common Covalent (Bio)­Conjugation Reactions for Functionalized ZIF-8 Nanoparticles

The strategy we employed is based on postsynthetic solvent-assisted ligand exchange (SALE), a method reported to efficiently exchange ligands in various MOFs, particularly those based on carboxylate ligands.^27,28^ In this work, we used this approach to introduce selected functional groups (–NH_2_, –CHO, and –COOH) onto the surface of ZIF-8. We then investigated whether these groups are accessible and can therefore be used in conjugation reactions to attach various molecules, enabling further functionalization of the surfaces of ZIF-8 nanoparticles for biomedical applications.

## Results and Discussion

### ZIF-8 Synthesis

Nanosized particles of ZIF-8 were prepared following a literature-reported procedure.^29^ Zinc nitrate hexahydrate was mixed with 2-methylimidazole in methanol, and the reaction was left undisturbed for 1 h at ambient temperature. A successful synthesis was confirmed by FTIR and XRPD measurements (Figures S1 and S2), as well as by STEM analysis, which revealed rhombic dodecahedral crystals with an average diameter of 87 ± 18 nm (Figure S3).

### Functionalizing ZIF-8 with Amino and Aldehyde Groups

Primary amino and aldehyde functional groups were introduced onto the particle surface using the postsynthetic SALE (solvent-assisted ligand exchange) approach. The nanocrystals were mixed with ligands bearing the corresponding functional groups in methanol at 60 °C. For ZIF-8–CHO, imidazole-2-carbaldehyde was used (Figure 2e), while for ZIF-8–NH_2_, 3-amino-1,2,4-triazole was chosen (Figure 2a) instead of 2-aminoimidazole due to its higher stability (storage at room temperature in air) and the previous work by Cho et al.,^30^ which showed that the original ZIF-8 structure is retained even after a majority of the imidazolate ligands have been replaced by the triazolate ligands.

**Figure 2:**
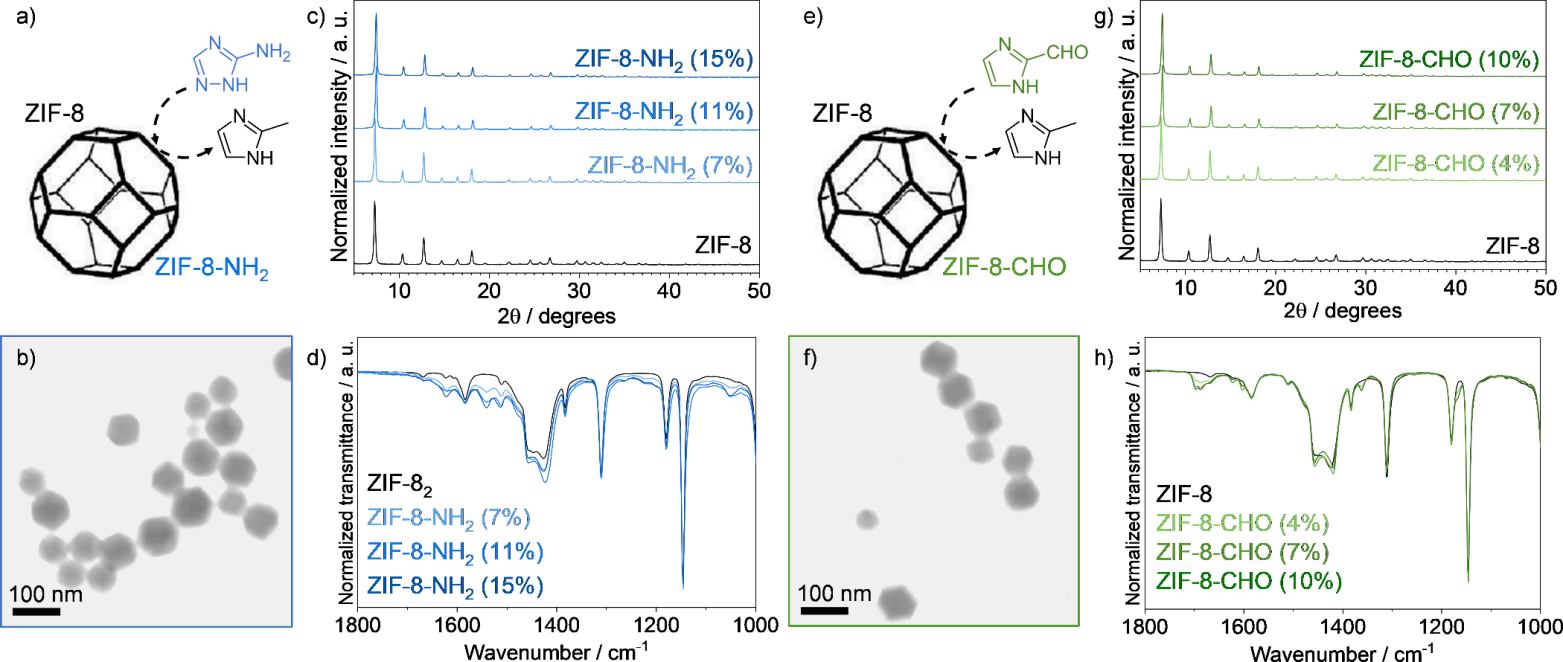
(a, e) Simplified reaction schemes of the ligand exchange, (b, f) STEM micrographs, (c, g) XRPD patterns, and (d, h) FTIR spectra of ZIF-8 functionalized with amino groups (ZIF-8–NH_2_, blue) and aldehyde groups (ZIF-8–CHO, green).

While it is desirable to exchange as few ligands as possible to retain the original properties of ZIF-8 particles, a certain minimum is required to cover the surface with functional groups. Because of the diffusion-controlled mechanism of SALE, ligand exchange begins at the surface and gradually progresses toward the inner layers of the MOF particles, as demonstrated by Jayachandrababu et al. using EDX mapping of partly exchanged micrometer-sized ZIF-8.^31^ Therefore, it can be assumed that functionalized ligands introduced by SALE are initially located in the outermost unit cells. For an idealized ZIF-8 single crystal with a midsphere diameter of 100 nm, approximately 8% of all unit cells lie at the surface; for a crystal with a midsphere diameter of 80 nm, this fraction increases to approximately 11%. Using the SALE approach, we showed that the degree of ligand exchange can be controlled by adjusting the reaction time, allowing for different levels of surface functionalization to be achieved. As a proof of principle, three samples with different degrees of exchange were prepared for both ZIF-8–NH_2_ and ZIF-8–CHO (Table 2).

**Table 2:**
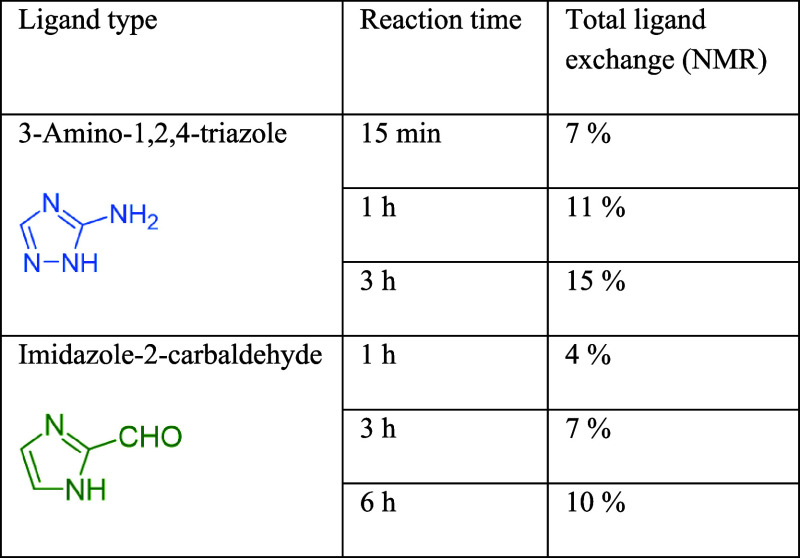
Overview of the Reaction Conditions Leading to Different Levels of Total Ligand Exchange as Determined by ^1^H NMR (Figures S4 and S5)

To quantify the degree of exchange, ^1^H NMR spectra of the acid-digested particles were recorded. These spectra were used to determine the ratio of the functionalized ligands to the original ligands (Figures S4 and S5) by comparing the integrated areas of signals corresponding to the aromatic protons of the 2-methylimidazole ligand at 7.45 ppm with those of the functionalized ligands at 8.3 ppm for ZIF-8–NH_2_ and 7.5 ppm for ZIF-8–CHO. The exact degree of exchange strongly depends on the type of functionalized ligand, as SALE proceeds faster with the amino-functionalized ligand than with the aldehyde derivative. For ZIF-8–NH_2_, reaction times of 15 min, 1 h, and 3 h yielded approximately 7, 11, and 15% exchange, respectively, whereas for ZIF-8–CHO, reaction times of 1, 3, and 6 h were required to achieve comparable exchanges of approximately 4, 7, and 10%, respectively (Table 2). The apparent slowdown of ligand exchange for both systems with increasing time is likely attributed to a transition from a rapid surface-dominated exchange regime to a diffusion-limited process. A similar kinetic trend was previously observed by Cho et al.,^30^ who investigated ligand exchange of 3-amino-1,2,4-triazole on ZIF-8 over extended reaction times of up to 48 h.

While XRPD patterns remained unchanged (Figure 2c,g), indicating that the crystal structure was stable under the SALE reaction conditions, successful integration of the functional groups was confirmed by FTIR spectroscopy, where new bands appeared compared to pristine ZIF-8 (Figure 2d,h and Figure S6).

In the FTIR spectra of ZIF-8–NH_2_ (Figure 2d and Figure S6), N–H vibrations of primary and secondary amino groups appear at 3460, 3320, and 1620 cm^–1^ and at 1050 cm^–1^, while C–N vibrations from the triazole moieties can be observed at 1540 and 1510 cm^–1^. For ZIF-8–CHO (Figure 2h and Figure S6), only the ν­(C=O) vibration of the carbonyl group emerges at 1690 cm^–1^. STEM analysis revealed that the particle morphology remained unchanged in all functionalized samples (Figure 2a,e and Figures S7 and S8). Moreover, nitrogen sorption analysis at 77 K confirmed that the samples remained porous, with BET surface areas of 1770 m^2^·g^–1^ for ZIF-8–NH_2_ (7%) and 1584 m^2^·g^–1^ for ZIF-8–CHO (7%) (Figure S9), which are comparable to that of pristine ZIF-8 (1784 m^2^·g^–1^, Figure S9).

### Functionalizing ZIF-8 with Carboxylic Acid Groups

Another functional group often used in conjugation reactions, for example, in carbodiimide coupling (Table 1), is the carboxylic acid group. While carboxylate ligands are commonly used to construct MOFs, carboxylic acid groups are absent in imidazolate MOFs, such as ZIF-8. Our attempts to introduce these groups in the same manner as the amine and aldehyde functional groups were unsuccessful (Figure 3a) because of the limited solubility of imidazole-2-carboxylic acid in methanol. However, it is known that glutaric anhydride can be used to convert primary amino groups to carboxylic acids^32^ by forming a stable amide bond upon opening the anhydride ring (Figure 3b). This approach would simultaneously give access to ZIF-8 functionalized with carboxylate groups (ZIF-8–COOH) and prove the addressability of the primary amino groups of ZIF-8–NH_2_ for covalent conjugation reactions.

**Figure 3:**
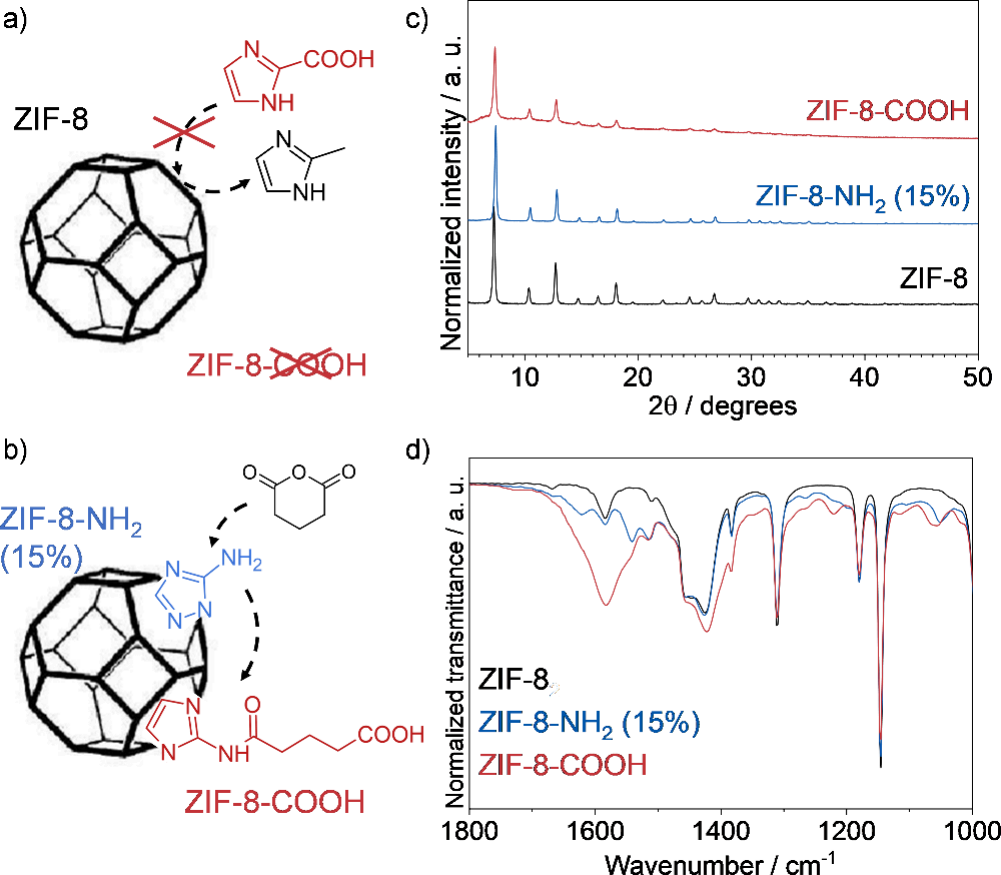
(a, b) Simplified reaction schemes for the synthesis of ZIF-8–COOH and (c) XRPD patterns and (d) FTIR spectra of ZIF-8, ZIF-8–NH_2_, and the product obtained after its functionalization with glutaric anhydride (ZIF-8–COOH).

The reaction was carried out on the 15% exchanged sample of ZIF-8–NH_2_. After stirring ZIF-8–NH_2_ in a solution of glutaric anhydride in anhydrous acetonitrile at room temperature for 24 h, a broad carbonyl band appeared at 1577 cm^–1^ in the FTIR spectrum (Figure 3d and Figure S10), indicating the formation of new amide and carboxylic acid moieties. While the XRPD pattern (Figure 3c) remained unchanged, minor contrast changes in the nanoparticles were observed in the STEM micrographs (Figure S11), suggesting partial etching of the crystals, although the overall crystal morphology remained intact. To further confirm and quantify the conversion, ^1^H NMR spectra of acid-digested particles were recorded (Figure S12). By comparison of the integrals of the aromatic signal of the aminotriazole with those of the signals corresponding to glutaric acid, 95% substitution of the amino groups was determined. In the NMR spectrum, the aminotriazole-glutaric acid conjugate was not observed; instead, only free aminotriazole and glutaric acid were detected. This is attributed to the harsh acidic conditions (32% DCl) used for crystal digestion, which cleaved the amide bonds. This hypothesis was confirmed using a reference sample prepared by reacting 3-amino-1,2,4-triazole with glutaric anhydride (Figure S13) and recording its NMR spectrum 1 h and 1 day after the addition of DCl. Whereas, without the addition of DCl, only the conjugate was detected; after 1 h, both the conjugate and the free components were observed; and after 1 day, only the free components were present (Figure S14).

### Demonstrating Chemical Accessibility of the Introduced Functional Groups

To demonstrate the accessibility of amino and aldehyde groups on the surface of ZIF-8, we investigated their conjugation with fluorescein-based dyes (Figure 4). Fluorescein has a van der Waals radius of approximately 7 Å,^33^ which is significantly larger than the pore aperture of ZIF-8 (3.4 Å)^1^ and, therefore, cannot penetrate the framework. However, it is worth noting that one should be cautious when selecting molecules for exclusive surface functionalization because several studies, both experimental and theoretical, have reported gate-opening behavior in ZIF-8,^34–38^ allowing molecules larger than the nominal pore opening (e.g., benzene,^39^ SF_6_,^40^ etc.) to enter the pores.

**Figure 4:**
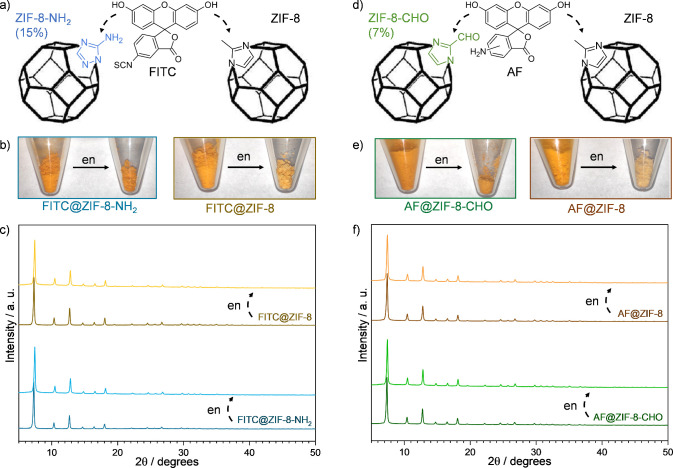
(a, d) Simplified reaction schemes for the reactions with fluorescein isothiocyanate (FTIC) and aminofluorescein (AF), (b, e) photographs of the samples before and after washing cycles with ethylenediamine (en), and (c, f) their XRPD patterns.

To functionalize the external surface of ZIF-8–NH_2_, a reaction with bromo-functionalized terpyridine in the presence of triethylamine has been reported recently.^41^ In this study, with regard to the focus on medical applications, we employed commercially available amine-reactive fluorescein isothiocyanate (FITC), which forms stable thiourea bonds without requiring additional reagents (Table 1). ZIF-8–CHO was reacted with aminofluorescein (AF) to enable a Schiff base reaction between the aldehyde and primary amine (Table 1). To stabilize the acid-sensitive imine bond, NaBH_3_CN was added to selectively reduce it to a more stable amine.^42^

Both dyes were reacted with their corresponding functionalized ZIF-8 counterparts and with pristine ZIF-8 as a negative control. STEM micrographs revealed no changes in the crystal morphology (Figure S15). All tests yielded bright orange solids without visible differences between functionalized and pristine ZIF-8 samples (Figure 4b,e). This can be explained by the fluorescein dyes being adsorbed on the particle surface via noncovalent interactions and/or coordination. Simple washing of the samples with various solvents (methanol, ethanol, water, dichloromethane, and toluene) did not sufficiently remove the noncovalently adsorbed dyes, so a solution of competitive chelating ligands, such as ethylenediamine (en), was used. At a concentration of 50 mM in ethanol, this treatment was capable of removing a significant amount of noncovalently bound dye within three incubation cycles of 50 min each, without damaging the particles. XRPD analysis confirmed that the samples remained crystalline after treatment with 50 mM en (Figure 4c,f). While the dyes were visibly removed from the nonfunctionalized ZIF-8 particles, which now appeared pale orange, they remained attached to the functionalized ZIF-8 particles, resulting in an unchanged bright orange color (Figure 4b,e). To quantify the amount of surface-bound dye before and after treatment with an ethylenediamine solution, a portion of each sample was digested in 1 M HCl, and the released fluorescein dye was quantified by UV–vis spectroscopy (Scheme S1 and Table S1 and Figure S16). The treatment resulted in a decrease in absorbance at the absorption maximum of only approximately 7.5% for FITC@ZIF-8–NH_2_, compared to 17.5% for FITC@ZIF-8. Similarly, AF@ZIF-8–CHO exhibited a decrease in absorbance of only 19.5%, whereas AF@ZIF-8 showed a substantially larger decrease of 73.6%. These findings indicate enhanced dye retention in the functionalized materials.

The performed experiments demonstrate the success of the covalent conjugation reactions and confirm the accessibility of the functional groups on the surfaces of ZIF-8–NH_2_ and ZIF-8–CHO. Moreover, they demonstrate a simple and efficient strategy for the covalent attachment of fluorescent dyes to the surfaces of ZIF-8 nanoparticles, which is highly attractive for biomedical applications.

To demonstrate the accessibility of the carboxylate group, ZIF-8–COOH was activated using 1-ethyl-3-(3-(dimethylamino)­propyl)­carbodiimide (EDC) and *N*-hydroxysuccinimide (NHS), followed by reaction with 2,2,2-trifluoroethylamine (Figure 5a). 2,2,2-Trifluoroethylamine was selected as a model amine due to its high volatility, which enables easy and complete removal of unreacted amine, and because it can be readily detected by ^19^F NMR spectroscopy. After conjugation, a new quartet appeared in the ^1^H NMR spectrum of an acid-digested sample at 3.8 ppm, which was absent in the negative control sample reacted with 2,2,2-trifluoroethylamine without prior carbodiimide activation (Figure S17). This quartet can be attributed to the CH_2_ protons of 2,2,2-trifluoroethylamine coupling to the adjacent fluorine nuclei. The corresponding signal is also observed in the ^19^F NMR spectrum of the activated ZIF-8–COOH, whereas it is absent in the control sample (Figure S18). The successful functionalization of NHS-activated ZIF-8–COOH with 2,2,2-trifluoroethylamine was further confirmed by FTIR (Figure 5c and Figure S19), and the retention of the sample crystallinity was verified by XRPD (Figure 5b). These results confirm the successful activation of the carboxyl groups and demonstrate the addressability of ZIF-8–COOH for conjugation reactions.

**Figure 5:**
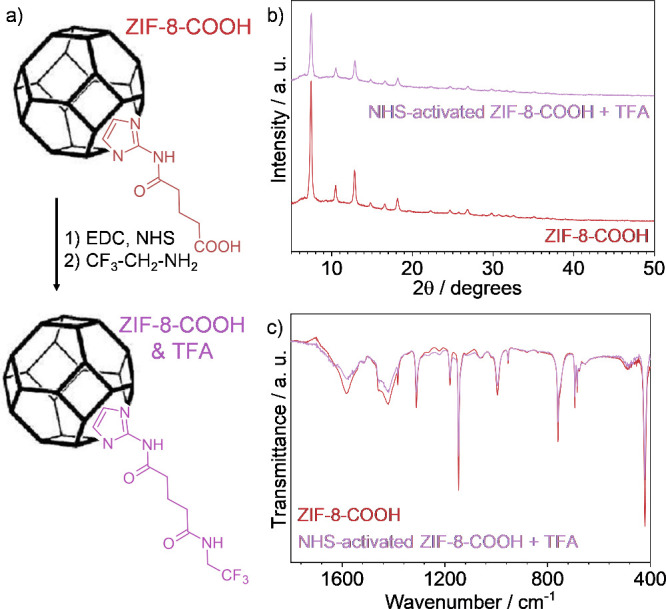
(a) Simplified reaction scheme for the conjugation of ZIF-8–COOH with 2,2,2-trifluoroethylamine (TFA) and the corresponding (b) XRPD patterns and (c) FTIR spectra.

## Conclusions

In this study, we report approaches for the successful functionalization of the external surfaces of ZIF-8 nanoparticles with three different functional groups: amino, aldehyde, and carboxylic acid groups, which are commonly used for (bio)­conjugation reactions. We demonstrated that the amount of functionalized ligands in the final material can be readily controlled by adjusting the reaction time, allowing for tuning of the material properties according to the intended application. Importantly, we confirmed that these functional groups are accessible and can participate in conjugation reactions to further modify the particle surfaces. For example, commercially available fluorescent dyes, such as fluorescein isothiocyanate and aminofluorescein, can be covalently attached to ZIF-8 nanoparticles, imparting imaging functionality that is valuable for *in vitro* biological studies. In the field of MOFs for biomedical applications, where surface chemistry critically influences material performance, the strategies presented here provide powerful tools to optimize ZIF-8 as a versatile platform for diverse applications.

## Experimental Section

### Materials and Methods

All reagents were of analytical grade and used as received from commercial suppliers. Fourier transform infrared (FTIR) spectra were recorded in the range of 400–4000 cm^–1^ on a Bruker Equinox 55 FTIR spectrometer equipped with an ATR unit. X-ray powder diffraction (XRPD) data were collected using a Seifert XRD 3003 TT powder diffractometer with a Meteor1D detector or an Empyrean (PANalytical) diffractometer equipped with a Bragg–Brentano HD mirror and a PIXcel3D 2 × 2 detector, operating at room temperature with Cu Kα_1_ radiation (λ = 1.54187 Å). Nitrogen adsorption–desorption isotherms were measured at 77 K using a 3Flex gas adsorption analyzer (Micromeritics). Prior to measurement, the samples were activated under dynamic vacuum at 150 °C for 3 h. The specific surface areas were determined using the Brunauer–Emmett–Teller (BET) method. UV–vis spectra were recorded on a SPECORD 210 PLUS spectrophotometer (analytikjena) using cuvettes with an optical path length of 0.5 cm. STEM micrographs were recorded on a Crossbeam 550 microscope (Zeiss). Samples were prepared by depositing a drop of the crystalline product dispersed in ethanol onto carbon-coated copper grids (200 mesh) and dried in air. ^1^H and ^19^F NMR spectra were recorded on Varian 400 and Bruker Ascend Evo 400 spectrometers. For liquid NMR analysis of MOF samples, 10 mg of crystalline sample was dissolved in 50 μL DCl (32% in D_2_O) and diluted with 0.5 mL DMSO-*d*_6_.

### Synthesis

#### Synthesis of ZIF-8

Nanosized ZIF-8 particles were synthesized following a literature procedure.^29^ Zn­(NO_3_)_2_·6H_2_O (734 mg, 2.47 mmol) and 2-methylimidazole (811 mg, 9.88 mmol) were separately dissolved in 50 mL of methanol each, then combined and stirred at room temperature for 1 h. The resulting white precipitate was collected by centrifugation (5000 rpm, 25 min), washed three times with methanol, and dried in air. Typical yields were 180 mg per batch.

#### Synthesis of ZIF-8–NH_2_

A modified literature procedure for solvent-assisted ligand exchange was used to prepare ZIF-8–NH_2_.^30^ ZIF-8 nanoparticles (60 mg) were dispersed in 12 mL of methanol by 1 min of ultrasonication. 3-Amino-1,2,4-triazole (11.1 mg, 0.132 mmol) was added, and the suspension was stirred at 60 °C for 15 min, 1 h, or 3 h to achieve degrees of exchange of approximately 7, 11, and 15%. After cooling to room temperature, the solid was collected by centrifugation (6500 rpm, 15 min), washed three times with methanol, and dried in air.

#### Synthesis of ZIF-8–CHO

A procedure analogous to that used for ZIF-8–NH_2_ was applied to prepare ZIF-8–CHO via solvent-assisted ligand exchange. ZIF-8 nanoparticles (100 mg) were dispersed in 20 mL of methanol by 1 min of ultrasonication. Imidazole-2-carbaldehyde (20.0 mg and 0.21 mmol for 4 and 7% substitution; 40.0 mg and 0.42 mmol for 10% substitution) was added, and the suspension was stirred at 60 °C for 1, 3, or 6 h to achieve the desired degrees of exchange (4, 7, and 10%, respectively). After cooling to room temperature, the solid was collected by centrifugation (6500 rpm, 15 min), washed three times with methanol, and dried in air.

#### Synthesis of ZIF-8–COOH

ZIF-8-NH_2_ nanoparticles with 15% ligand exchange (50.0 mg) were dispersed in 12.5 mL of acetonitrile by 1 min of ultrasonication. Glutaric anhydride (50.0 mg, 0.438 mmol) was added, and the mixture was stirred at room temperature for 22 h. The solid was collected by centrifugation (6500 rpm for 15 min), washed three times with acetonitrile, and dried at room temperature.

#### Carbodiimide Activation of ZIF-8–COOH and Subsequent Conjugation with 2,2,2-Trifluoroethylamine

ZIF-8–COOH (30.0 mg) was dispersed in 2.0 mL of anhydrous dichloromethane. Diisopropylethylamine (12.0 μL, 0.069 mmol), *N*-hydroxysuccinimide (7.9 mg, 0.069 mmol), and 1-ethyl-3-(3-(dimethylamino)­propyl)­carbodiimide hydrochloride (13.2 mg, 0.069 mmol) were added, and the mixture was stirred at room temperature for 24 h. The solid was collected by centrifugation, washed three times with dichloromethane, and dried under vacuum at room temperature. The dried solid was then treated with 400 μL (5.1 mmol) of 2,2,2-trifluoroethylamine in a closed vial and stirred at room temperature for 15 h. As a control, the same amount of 2,2,2-trifluoroethylamine was added to ZIF-8–COOH without prior carbodiimide activation. Unreacted 2,2,2-trifluoroethylamine was removed by heating the samples in an oven at 60 °C overnight.

#### Synthesis of FITC@ZIF-8–NH_2_ and FITC@ZIF-8

The 50.0 mg of ZIF-8 nanoparticles (15% –NH_2_ or pristine) were dispersed in 5.0 mL of methanol, and fluorescein isothiocyanate (FITC, 1.0 mg, 2.6 μmol) was added. The suspension was agitated on an orbital shaker at 240 rpm at ambient temperature under exclusion of light for 24 h. The resulting bright orange solid was collected by centrifugation (6500 rpm, 15 min), washed three times with methanol to remove unreacted dye, and dried in air at ambient temperature.

#### Synthesis of AF@ZIF-8–CHO and AF@ZIF-8

The 150.0 mg of ZIF-8 nanoparticles (7% –CHO or pristine) were dispersed in 15.0 mL of methanol, and aminofluorescein (3.0 mg, 8.6 μmol) was added. The suspension was stirred at ambient temperature under exclusion of light for 1 h, followed by the addition of 6.0 mg (0.10 mmol) of sodium cyanoborohydride (NaBH_3_CN). Stirring was continued for an additional 23 h. The resulting bright orange solid was collected by centrifugation (6500 rpm, 15 min), washed three times with methanol to remove unreacted dye, and dried at ambient temperature.

#### Dye Desorption Tests

The 15.0 mg of dye-functionalized ZIF nanoparticles were dispersed in 3.75 mL of a 0.05 M solution of ethylene diamine (en) in ethanol. The suspension was maintained at ambient temperature for 50 min with occasional shaking to redisperse sedimented particles, followed by centrifugation (6500 rpm for 5 min). The supernatant was discarded, and the particles were redispersed in fresh en-solution for another 50 min. This procedure was repeated for a total of three times. The final particles were washed with pure ethanol and dried at ambient temperature in air.

## Supplementary Material



## Notes

NMR and FTIR spectra, STEM micrographs, and XRPD patterns (PDF)
