# Supplementary material for: Surface Functionalization of Zeolitic Imidazolate Framework‑8 Nanoparticles with Accessible Groups for Covalent Conjugation
Source: Inorg Chem. 2026 Jul 1;65(27):15697–704. doi: 10.1021/acs.inorgchem.6c01777 (PMC13370882; doi:10.1021/acs.inorgchem.6c01777)
Supplement: Supplementary file 1 [file ic6c01777_si_001.pdf]

Supporting Information

Surface Functionalization of ZIF-8 Nanoparticles with Accessible Groups for Covalent Conjugation

Michael B. Stammler[1], Hana Bunzen[1,2,*]

[1] Chair of Solid State and Materials Chemistry, Institute of Physics, University of Augsburg, Universitätsstraße 1, 86159 Augsburg, Germany
[2] Chair of Inorganic Chemistry, Institute of Chemistry, Otto-von-Guericke University Magdeburg, Universitätsplatz 2, 39106 Magdeburg, Germany; hana.bunzen@ovgu.de

Content:

1. Characterization of ZIF-8 nanoparticles S2
2. ^{1}H-NMR spectra of acid-digested samples of ZIF-8-NH_2 S4
3. ^{1}H-NMR spectra of acid-digested samples of ZIF-8-CHO S4
4. FTIR spectra of ZIF-8 crystals after solvent-assisted ligand exchange (-NH_2, -CHO) S6
5. STEM micrographs of ZIF-8 crystals after solvent-assisted ligand exchange (-NH_2, -CHO) S7
6. Sorption analysis S9
7. Characterization of ZIF-8-COOH S10
8. Conjugation reactions with fluorescein-based dyes S13
9. UV-Vis quantification of fluorescein content in samples of ZIF 8 derivatives S14
10. Conjugation reactions with 2,2,2-trifluoroethylamine S16

1. Characterization of ZIF-8 nanoparticles

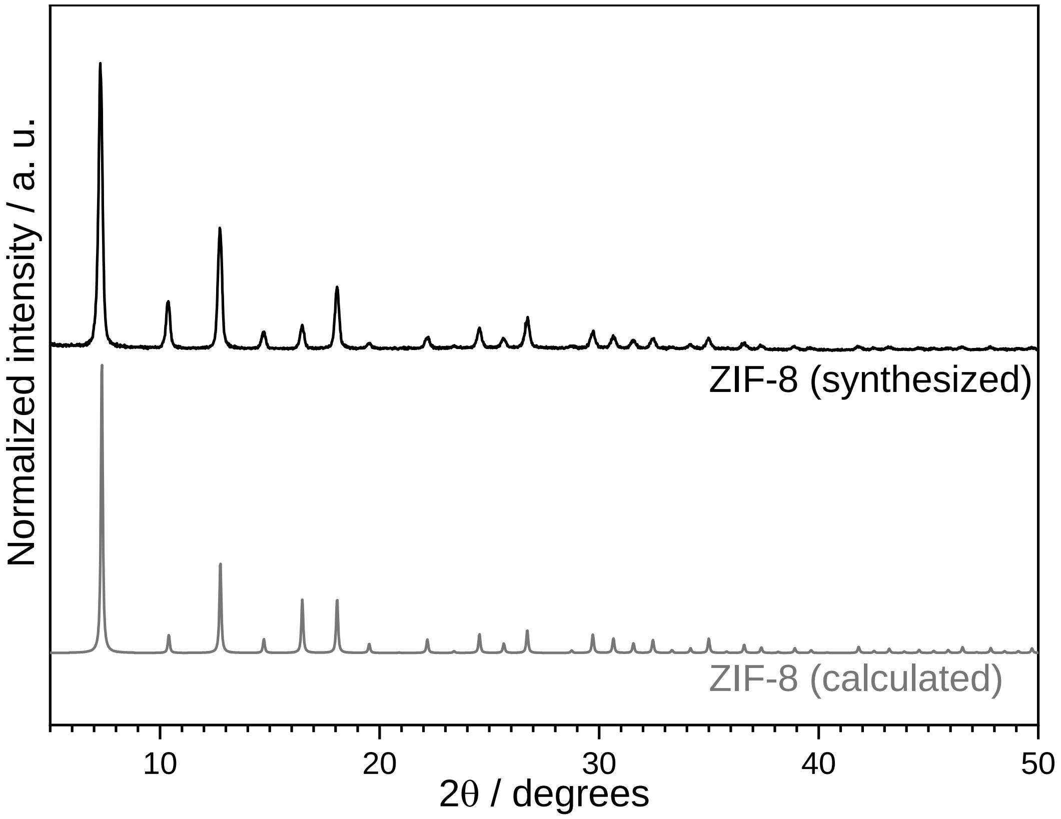

Figure S1. XRPD pattern of the synthesized ZIF-8 nanoparticles (black) and its comparison to the calculated data from single crystal X-ray diffraction analysis (grey, CCDC: 602542).

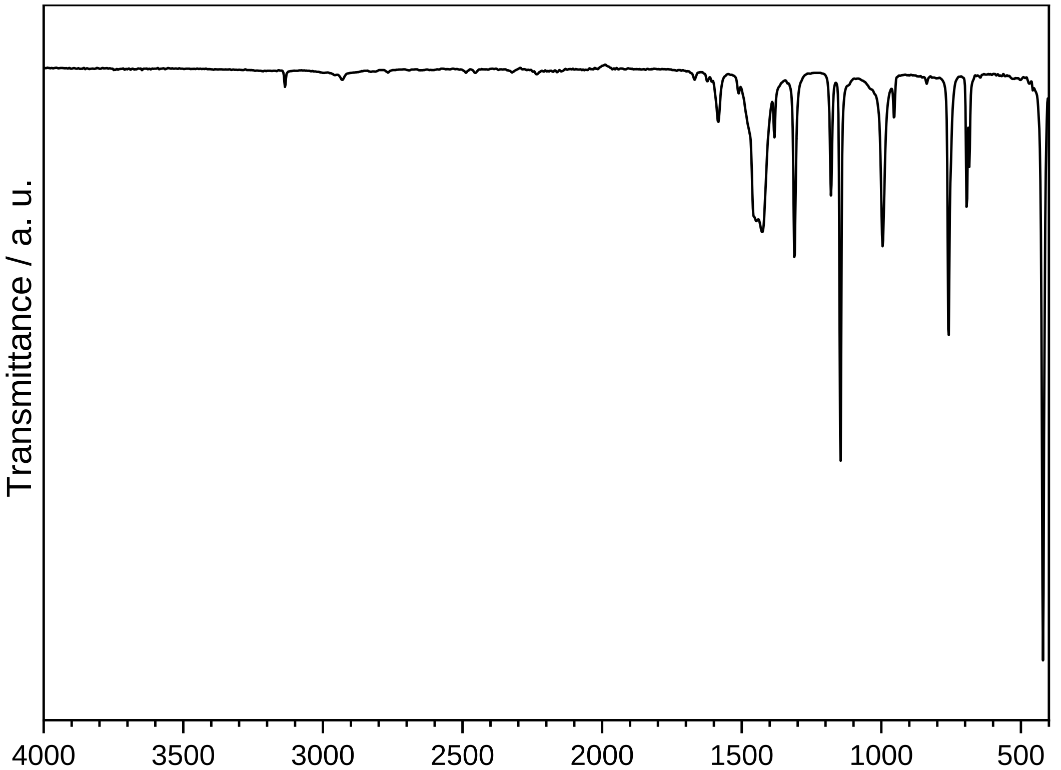

Figure S2. FTIR spectrum of the synthesized ZIF-8 nanoparticles.

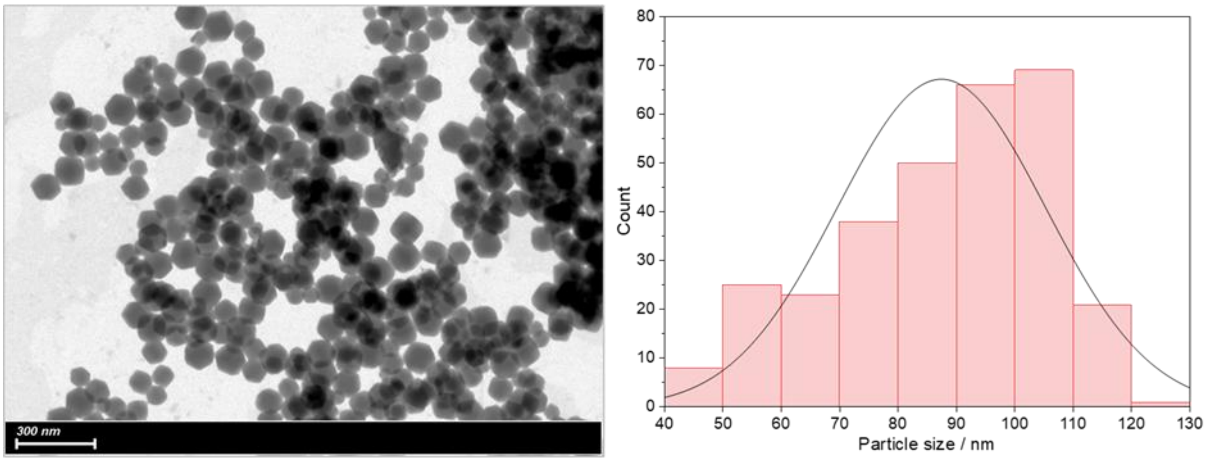

Figure S3. STEM micrograph and corresponding particle size histogram (based on the analysis of 300 particles) of the synthesized ZIF-8 nanoparticles.

2. ^{1}H-NMR spectra of acid-digested samples of ZIF-8-NH_2

(a)

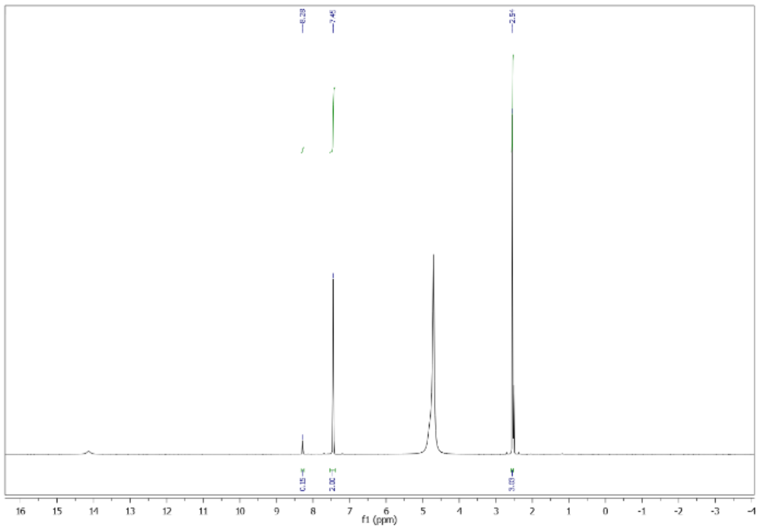

(b)

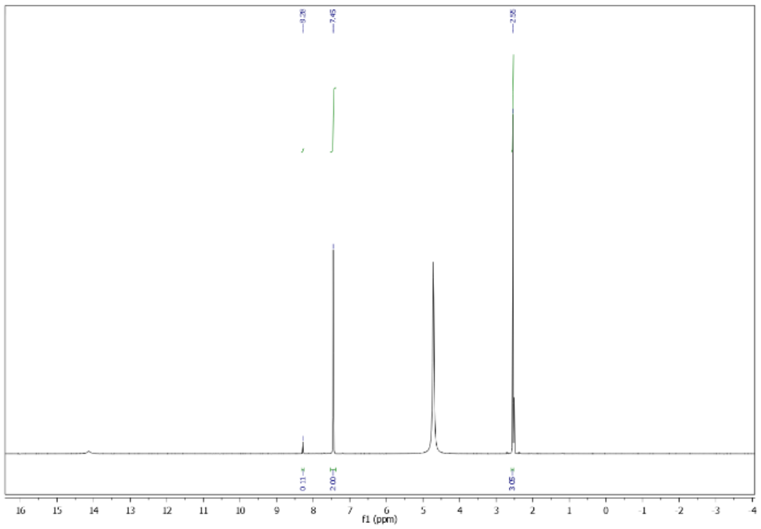

(c)

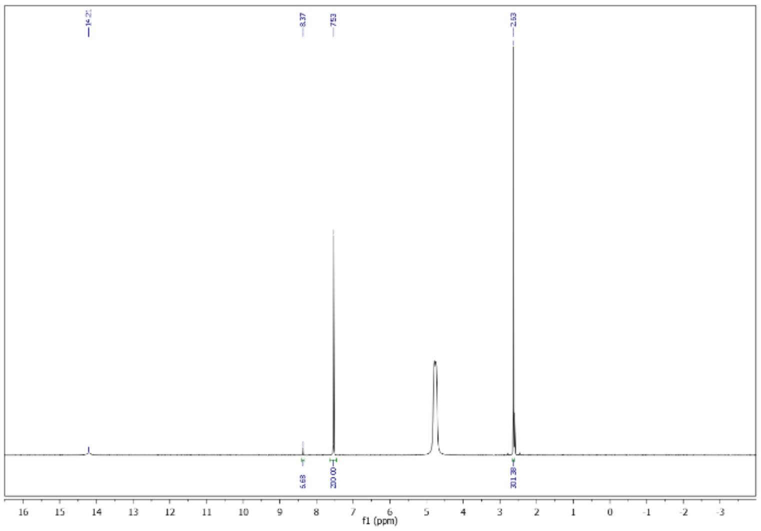

Figure S4. ^{1}H-NMR spectra of ZIF-8-NH_2 with ca. (a) 15% (b) 11 % and (c) 7 % linker exchange; DMSO-d_6:DCl = 10:1.

3. ^{1}H-NMR spectra of acid-digested samples of ZIF-8-CHO

(a)

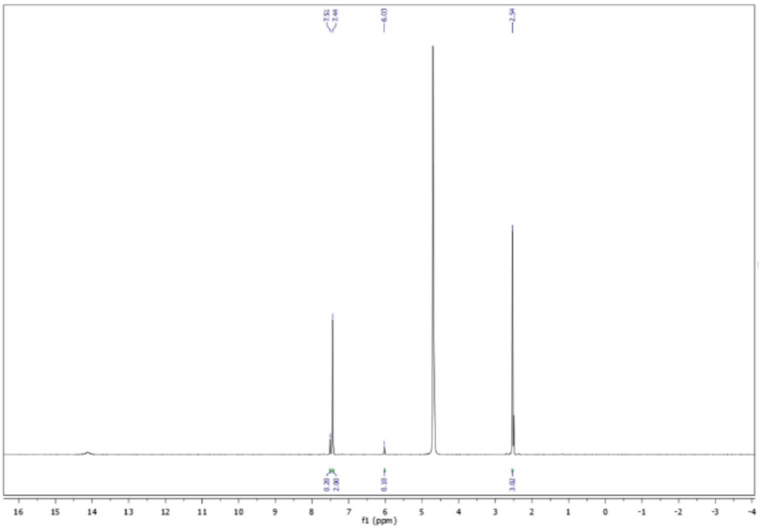

(b)

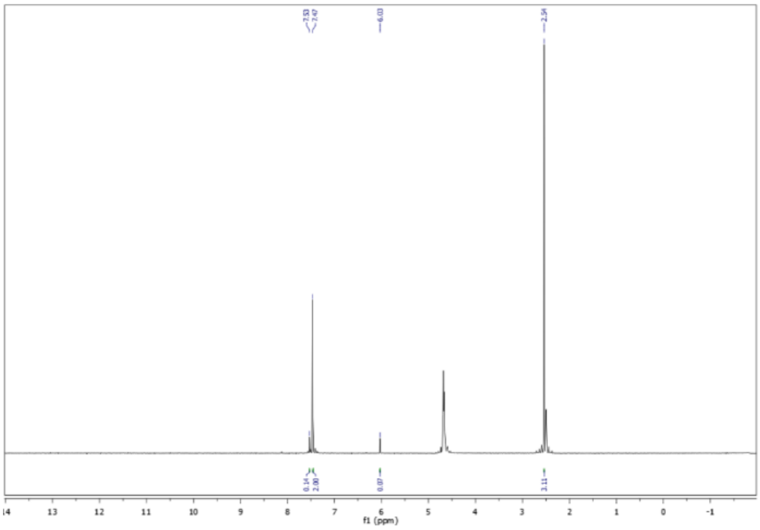

(c)

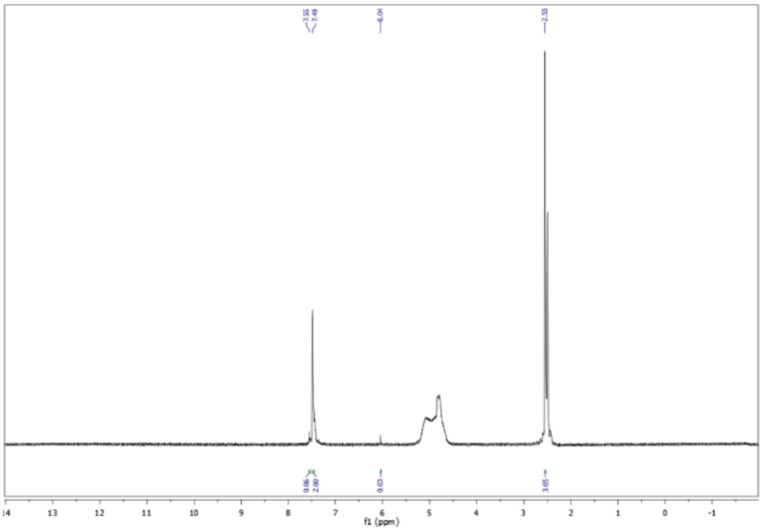

Figure S5. ^{1}H-NMR spectra of ZIF-8-NH_2 with ca. (a) 10% (b) 7 % and (c) 3 % linker exchange; DMSO-d_6:DCl = 10:1.

4. FTIR spectra of ZIF-8 crystals after solvent-assisted ligand exchange

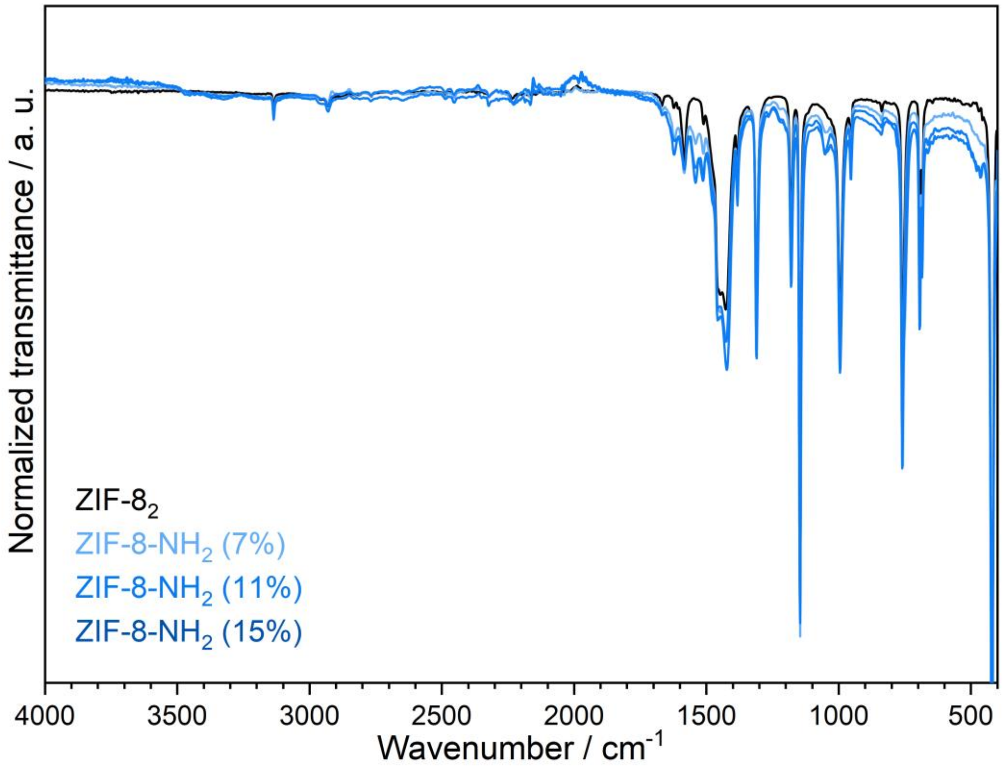

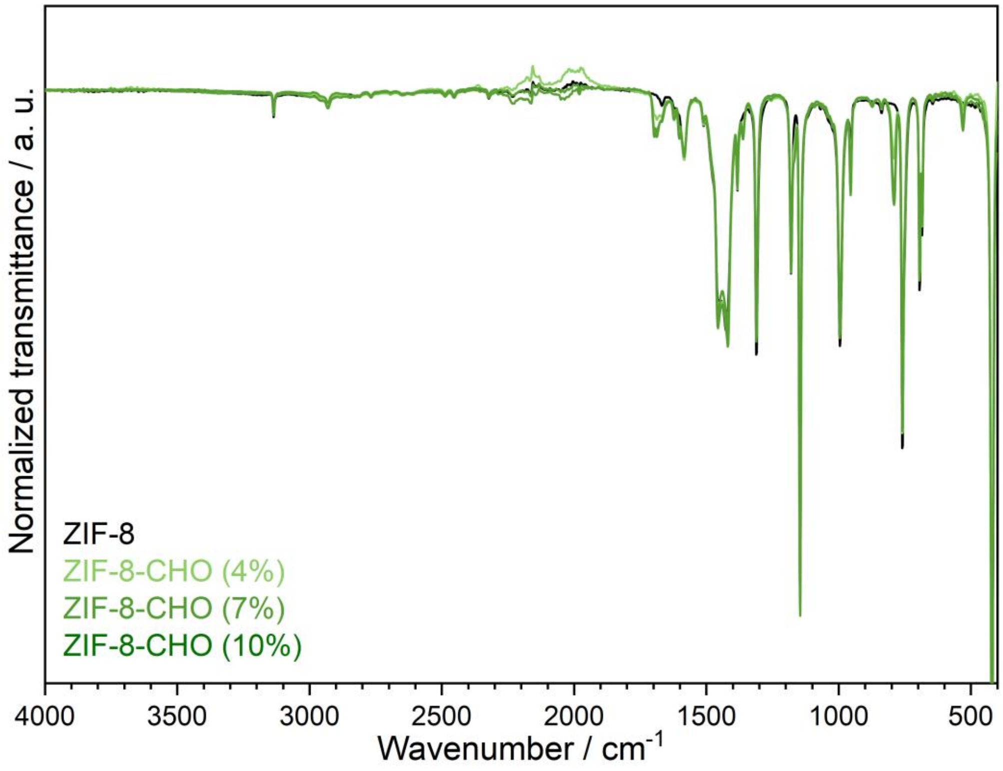

Figure S6. FTIR spectra of ZIF-8 functionalized with amino groups (ZIF-8-NH_2, top) and aldehyde groups (ZIF-8-CHO, bottom).

5. STEM micrographs of ZIF-8 crystals after solvent-assisted ligand exchange (-NH_2, -CHO)

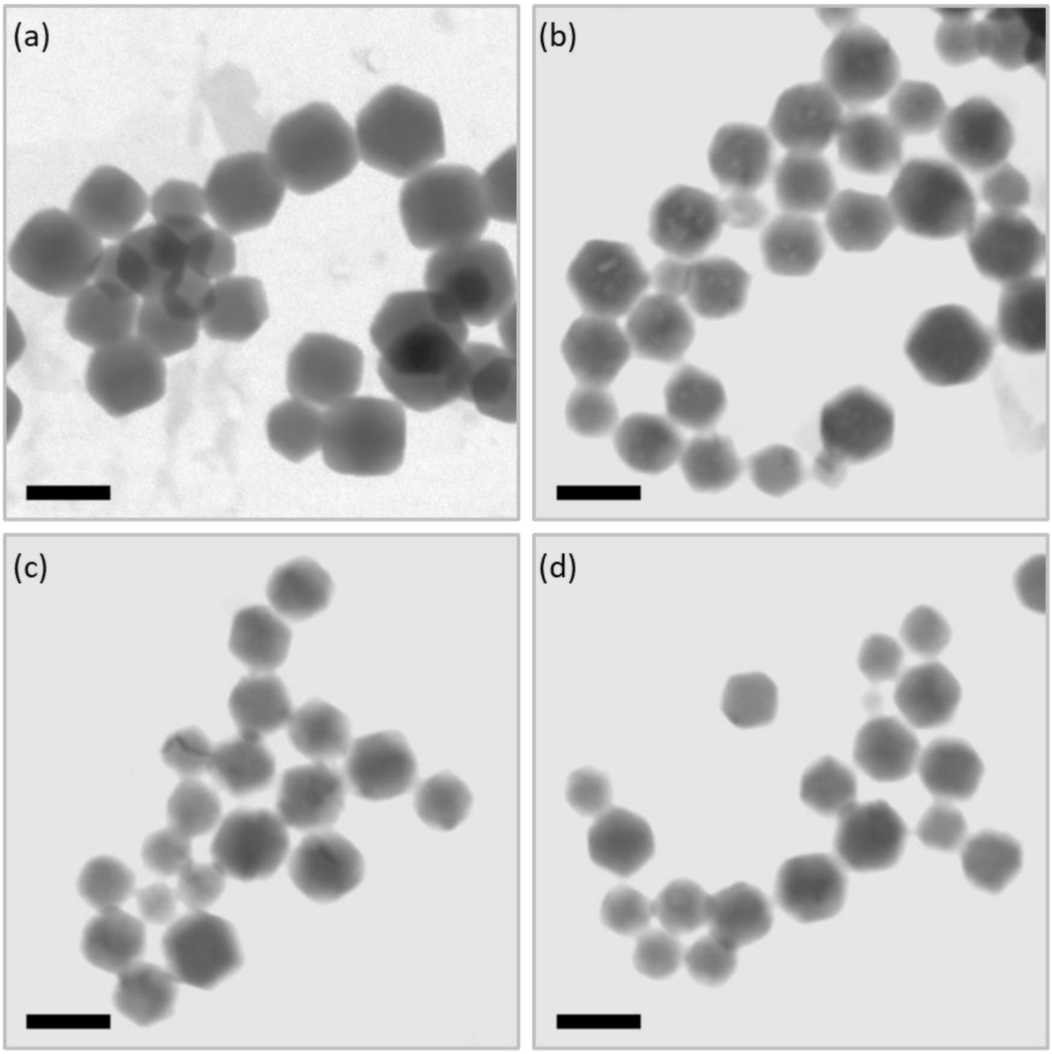

Figure S7. STEM micrographs of ZIF-8 (a) before and (b–d) after SALE with 3-amino-1,2,4-triazole, showing degrees of linker exchange of (b) 7%, (c) 11%, and (d) 15%. Scale bars equal 100 nm.

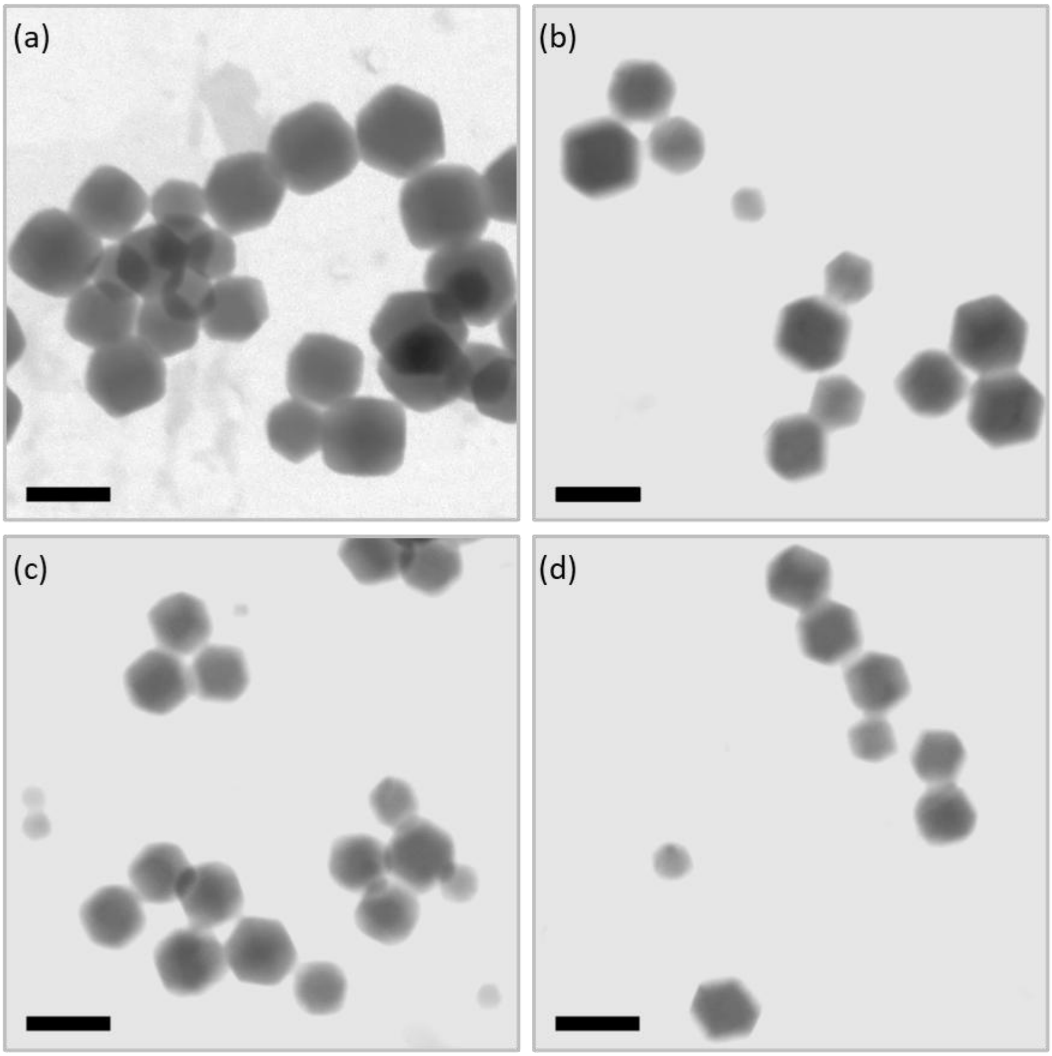

Figure S8. STEM micrographs of ZIF-8 (a) before and (b–d) after SALE with imidazole-2-carbaldehyde, showing degrees of linker exchange of (b) 4%, (c) 7%, and (d) 10%. Scale bars equal 100 nm.

6. Sorption analysis

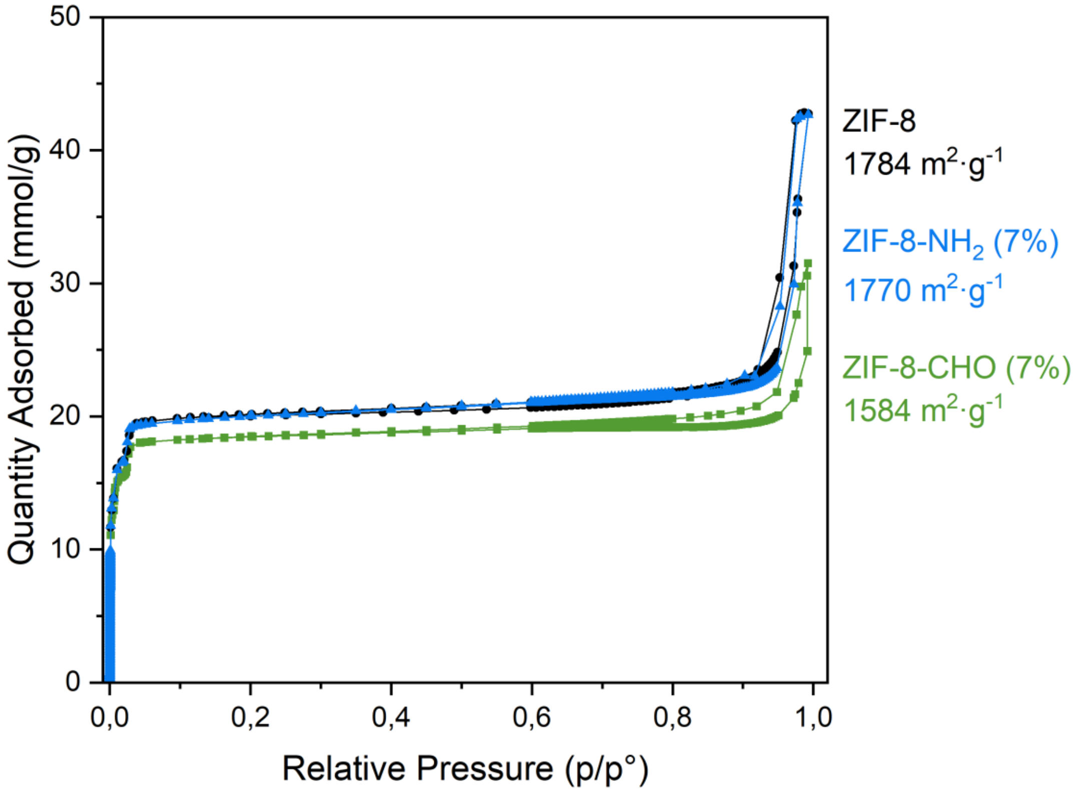

Figure S9. Nitrogen adsorption/desorption isotherms of ZIF-8 (black), ZIF-8-HN$_2$ (7%) (blue) and ZIF-8-CHO (7%) (green) measured at 77 K.

7. Characterization of ZIF-8-COOH

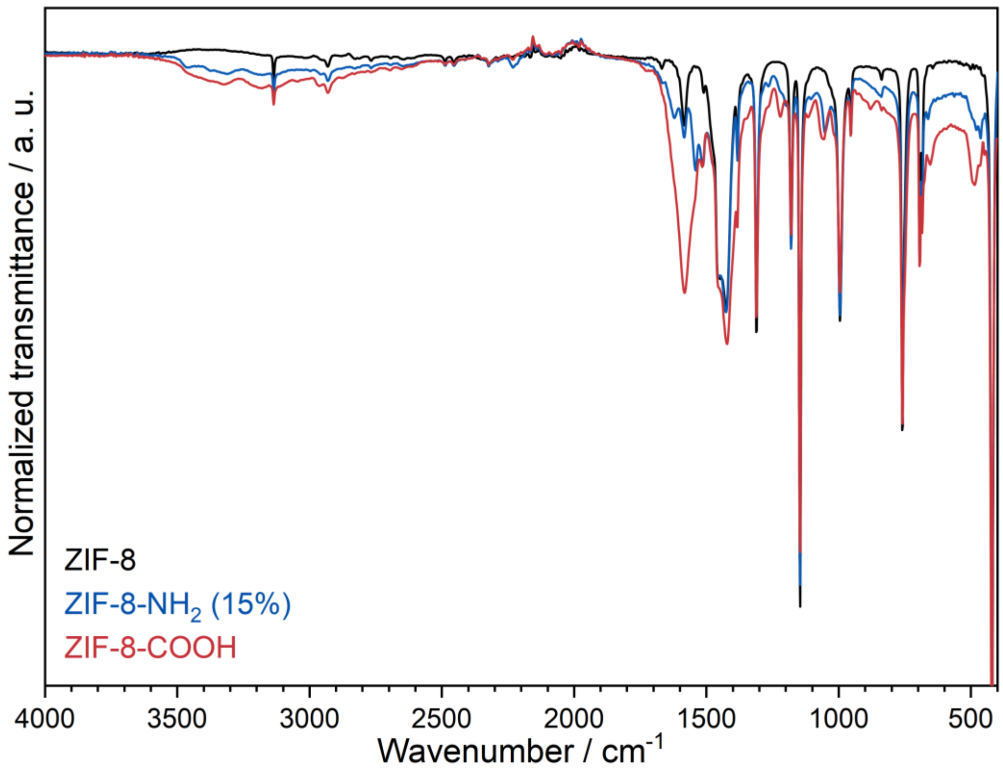

Figure S10. FTIR spectra of ZIF-8, ZIF-8-NH_2 and its product functionalized with glutaric anhydride (ZIF-8-COOH).

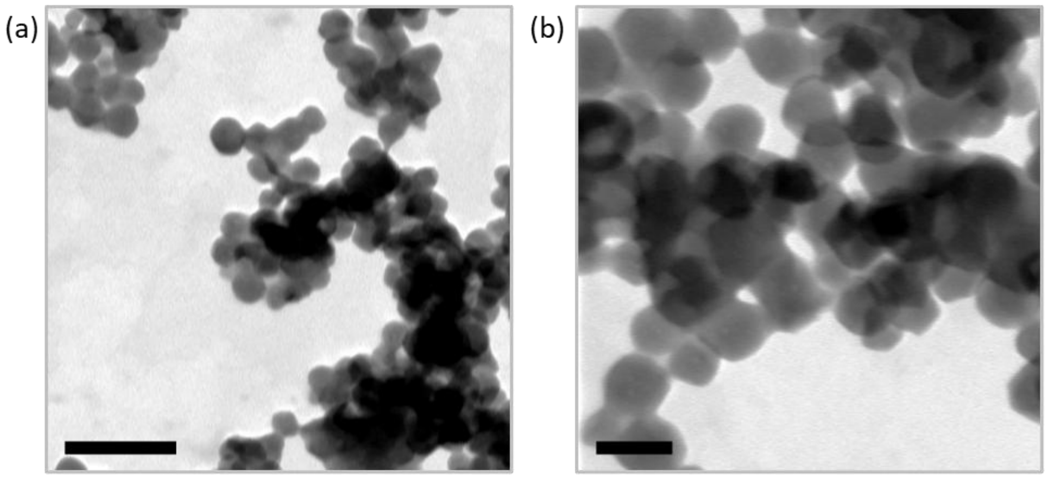

Figure S11. STEM micrographs of ZIF-8-COOH at magnifications of (a) 50k and (b) 100k. Scale bars equal (a) 300 nm and (b) 100 nm.

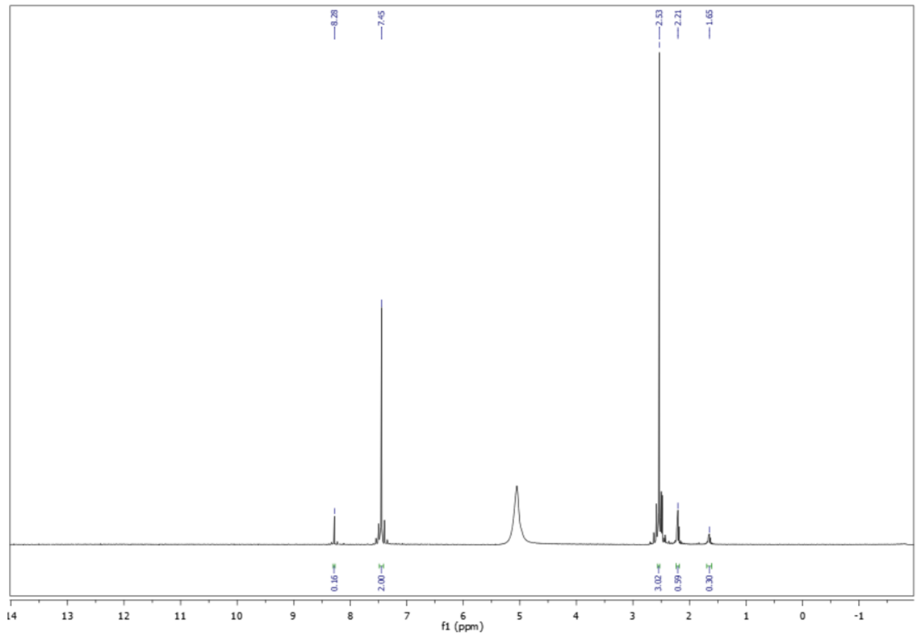

Figure S12. ^{1}H-NMR spectrum of ZIF-8-COOH (DMSO-d_6:DCl = 10:1).

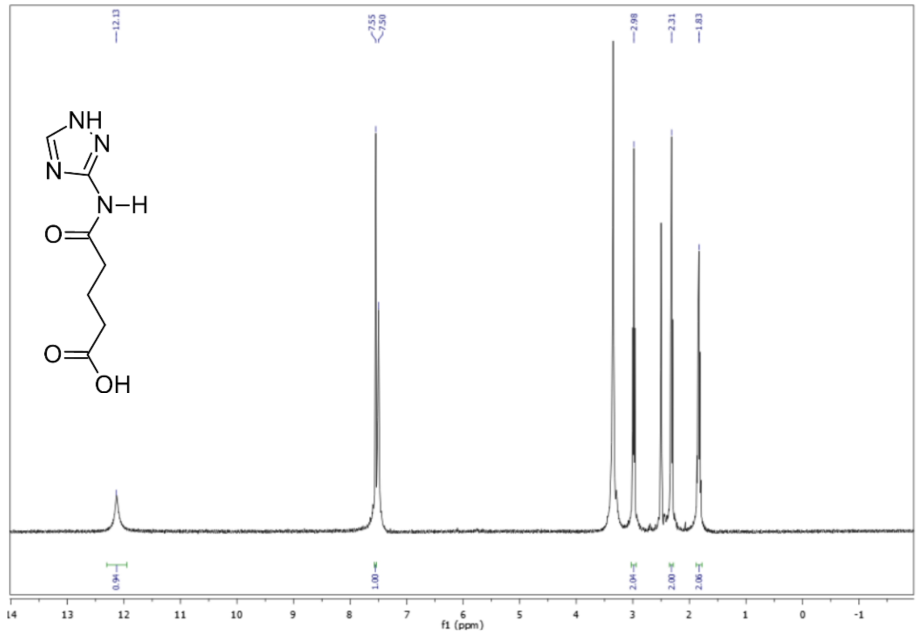

Figure S13. ^{1}H-NMR spectrum of the reaction product of 3-amino-1,2,4-triazole with glutaric anhydride (DMSO-d_6).

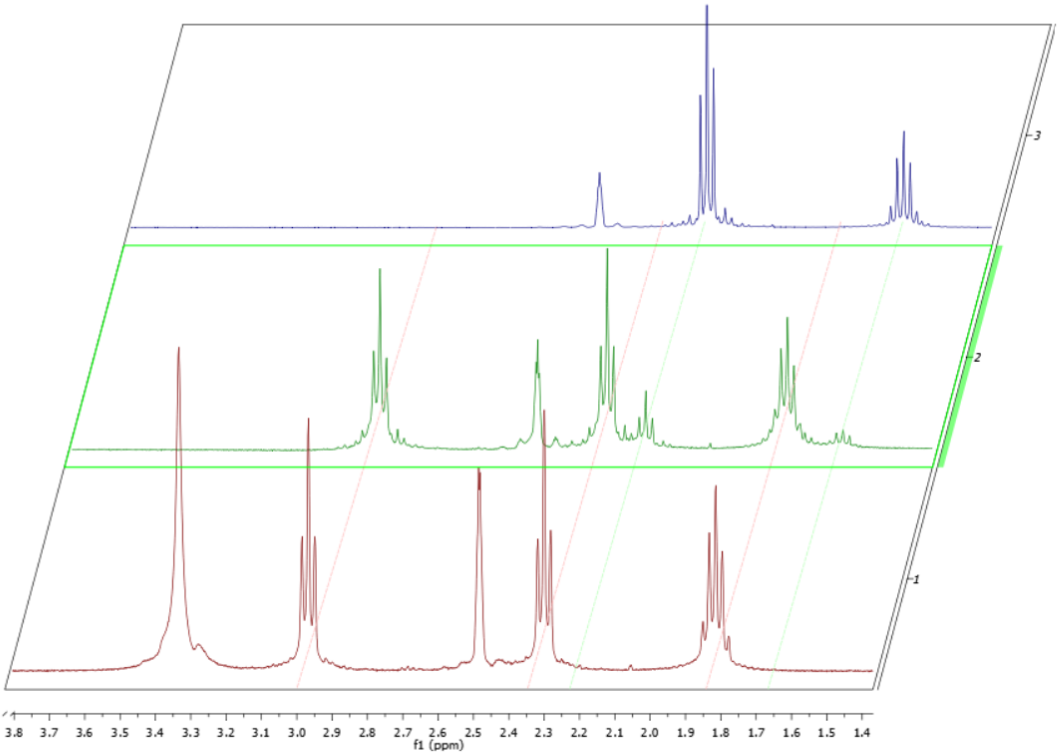

Figure S14. ^{1}H-NMR spectra of the reaction product of 3-amino-1,2,4-triazole with glutaric anhydride (1, red line), 1 h after the addition of DCl (2, green line), and 1 d after the addition of DCl (3, blue line) (DMSO-d_6).

8. Conjugation reactions with fluorescein-based dyes

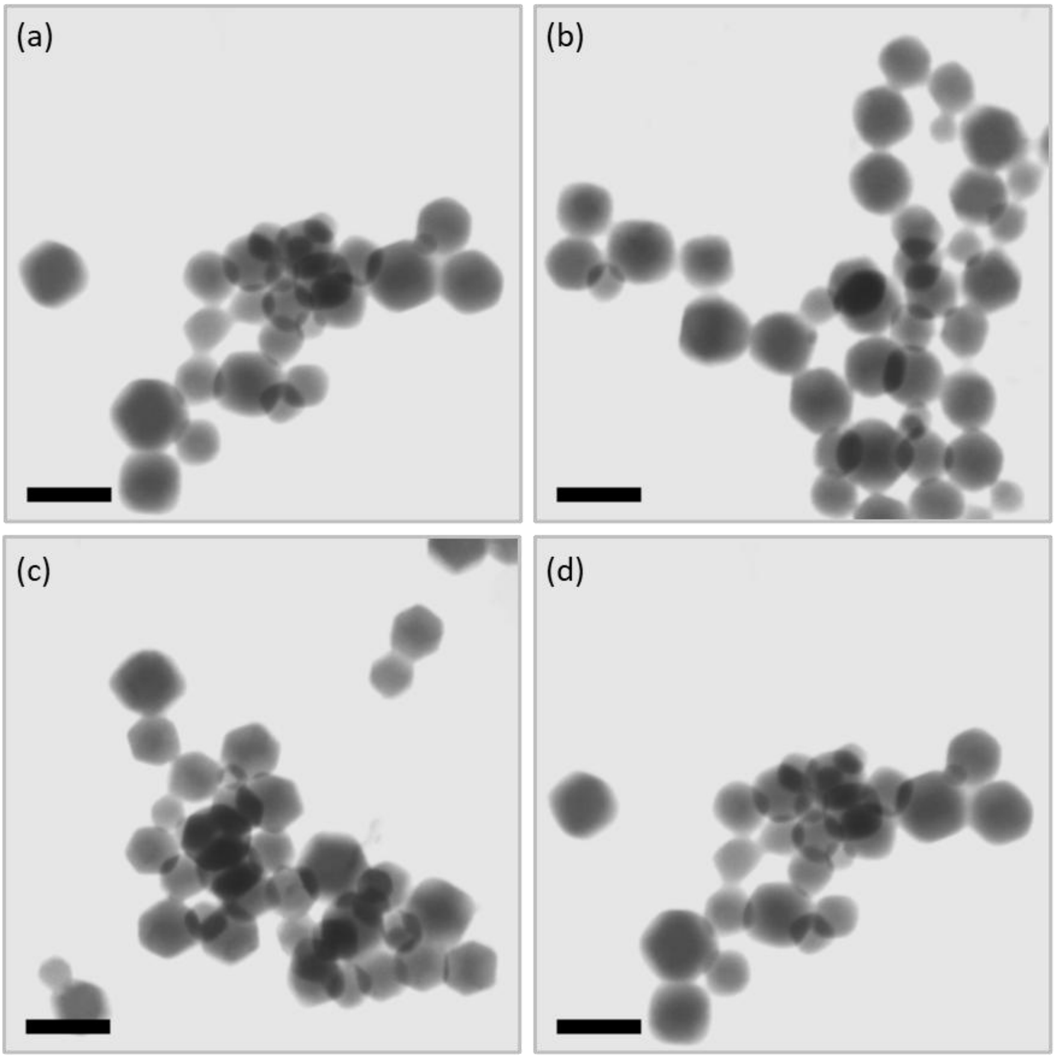

Figure S15. STEM micrographs of ZIF-8 samples conjugated with fluorescein-based dyes after treatment with ethylenediamine: (a) FITC@ZIF-8-NH_2, (b) FITC@ZIF-8, (c) AF@ZIF-8-CHO and (d) AF@ZIF-8. Scale bars equal 100 nm.

9. UV-Vis quantification of fluorescein content in samples of ZIF-8 derivatives

For the determination of the fluorescein content, 2.5 mg of each sample was dissolved in 5-6 drops of 1 M HCl. Subsequently, sufficient 0.01 M PBS (pH 7.4) was added to fully immerse the pH electrode in the solution. The pH was then adjusted to exactly 7.4 using 0.1 M NaOH. The resulting solution was transferred to a 25 mL volumetric flask and diluted to volume with PBS (0.01 M, pH 7.4). UV–Vis absorption spectra were recorded using a 0.5 cm path-length cuvette (Fig. S16). The absorbance maxima at 492 nm for FITC and 489 nm for AF were used to quantify changes in dye content before and after treatment with ethylenediamine (Table S1).

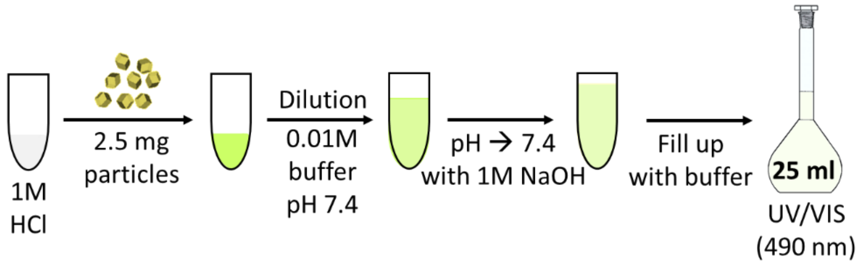

Scheme S1. Schematic presentation of the quantification of the fluorescein content in samples of ZIF-8 derivatives.

Table S1. Overview of the absorbance values of samples prepared according to the procedure shown in Scheme S1.

Sample	m_{MOF} / mg	V / ml	Absorbance maximum	Difference (before-after)
FITC@ZIF-8 (before treatment with en)	2.5	25	A_{492nm}=0.1900	-17.5%
FITC@ZIF-8 (after treatment with en)	2.5	25	A_{492nm}=0.1568	
FITC@ZIF-8-NH_2 (before treatment with en)	2.5	25	A_{492nm}=0.1934	-7.5%
FITC@ZIF-8-NH_2 (after treatment with en)	2.5	25	A_{492nm}=0.1788	
AF@ZIF-8 (before treatment with en)	2.5	25	A_{489nm}=0.1050	-73.6%
AF@ZIF-8 (after treatment with en)	2.5	25	A_{489nm}=0.0277	
AF@ZIF-8-CHO (before treatment with en)	2.5	25	A_{489nm}=0.0820	-19.5%
AF@ZIF-8-CHO (after treatment with en)	2.5	25	A_{489nm}=0.0660	

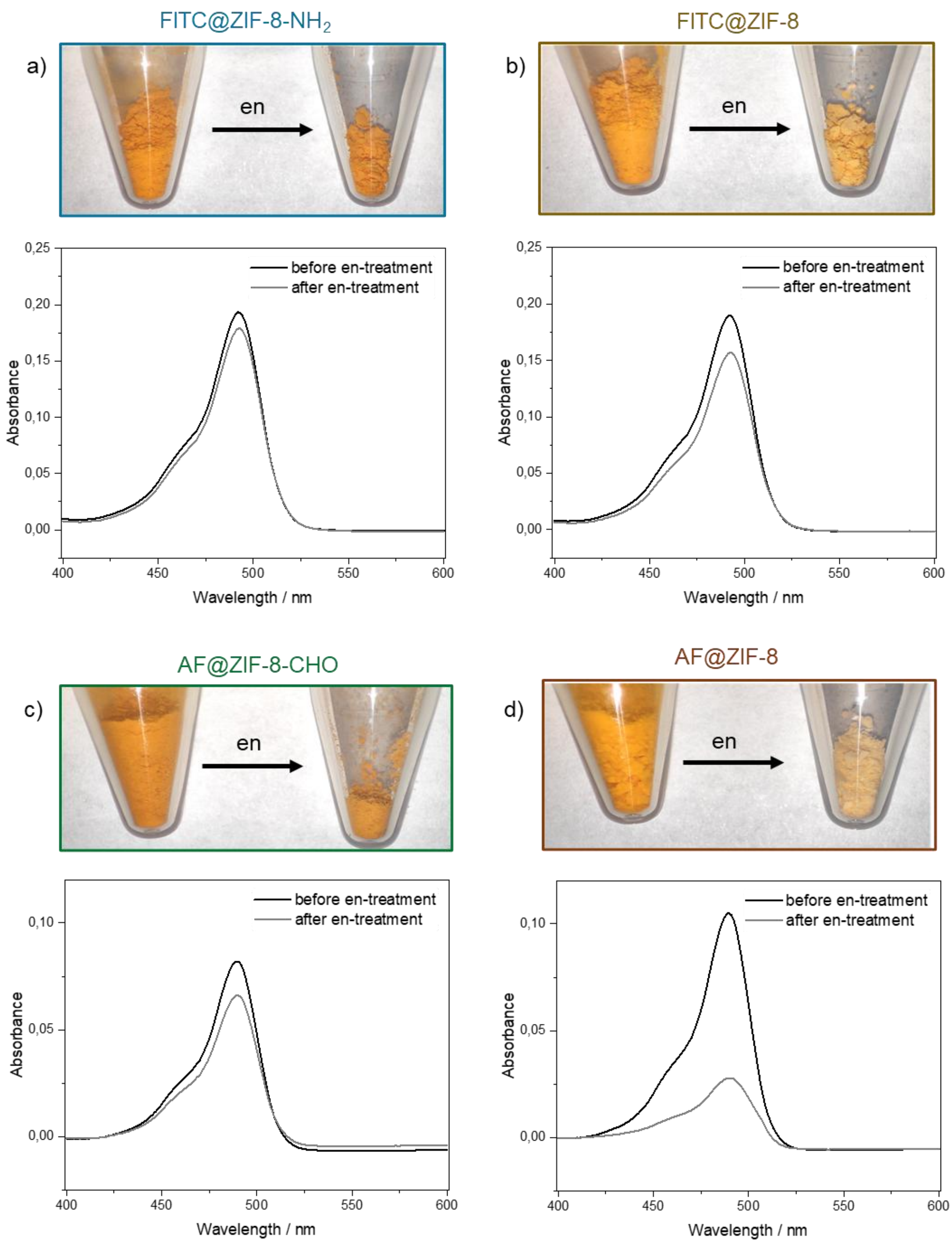

Figure S16. Photographs and UV–Vis spectra of the samples before and after washing cycles with ethylenediamine (en). A summary of the absorption maxima is given in Table S1.

10. Conjugation reactions with 2,2,2-trifluoroethylamine

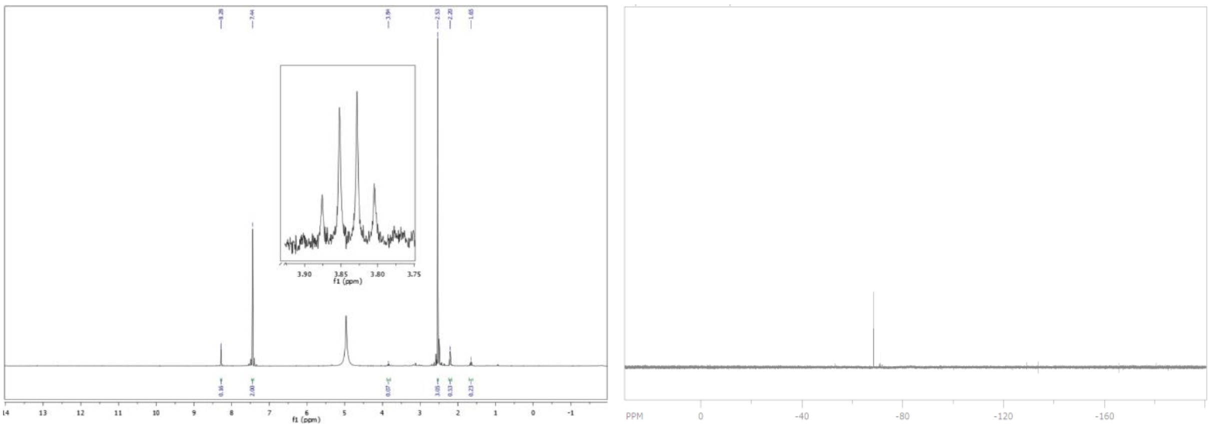

Figure S17. ^{1}H-NMR (left) and ^{19}F-NMR (right) spectra of the reaction product of NHS-activated ZIF-8-COOH with 2,2,2-trifluoroethylamine (DMSO-d_6:DCl = 10:1).

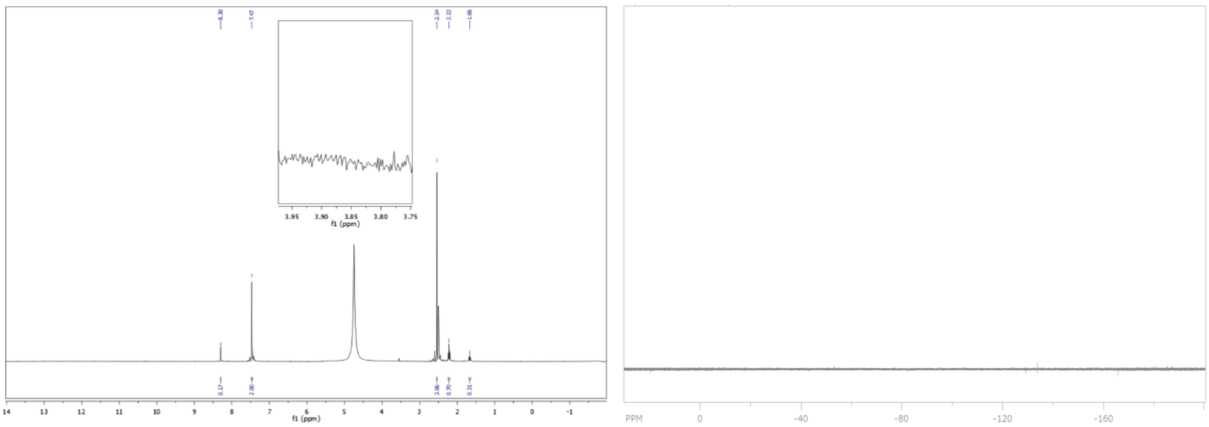

Figure S18. ^{1}H-NMR (left) and ^{19}F-NMR (right) spectra of the reaction product of ZIF-8-COOH (not NHS-activated) with 2,2,2-trifluoroethylamine (DMSO-d_6:DCl = 10:1).

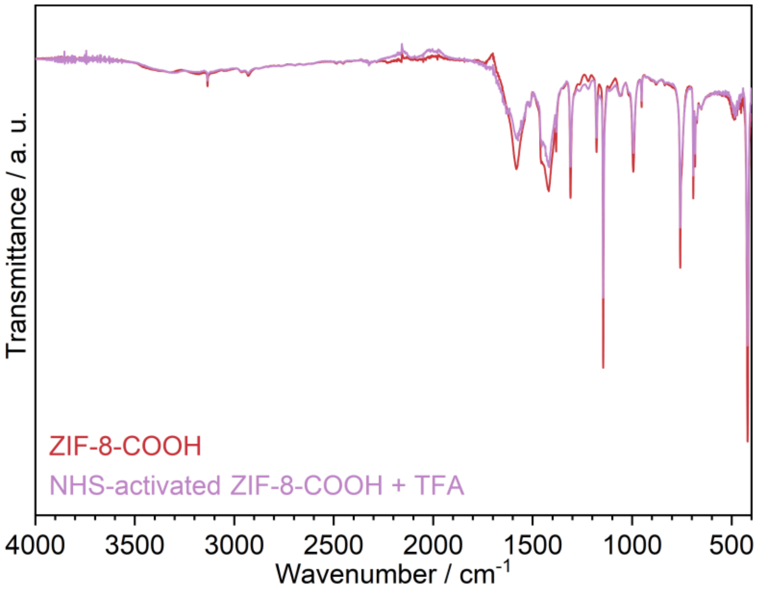

Figure S19. FTIR spectra of ZIF-8-COOH (red) and the reaction product of NHS-activated ZIF-8-COOH with 2,2,2-trifluoroethylamine (violet).
